# Soybean yield, biological N_2_ fixation and seed composition responses to additional inoculation in the United States

**DOI:** 10.1038/s41598-019-56465-0

**Published:** 2019-12-27

**Authors:** Walter D. Carciochi, Luiz H. Moro Rosso, Mario A. Secchi, Adalgisa R. Torres, Seth Naeve, Shaun N. Casteel, Péter Kovács, Dan Davidson, Larry C. Purcell, Sotirios Archontoulis, Ignacio A. Ciampitti

**Affiliations:** 1 Department of Agronomy, Kansas State University, Manhattan, KS US; 2 Department of Agronomy and Plant Genetics, University of Minnesota, St. Paul, MN US; 3 Department of Agronomy, Purdue University, West Lafayette, IN US; 4 Department of Agronomy, Horticulture, and Plant Science, South Dakota State University, Brookings, SD US; 5 Illinois Soybean Association, Bloomington, IL US; 6 Department of Crop, Soil, and Environmental Sciences, University of Arkansas, Fayetteville, AR US; 7 Department of Agronomy, Iowa State University, Ames, IA US

**Keywords:** Biotechnology, Plant sciences

## Abstract

It is unclear if additional inoculation with *Bradyrhizobia* at varying soybean [*Glycine max* (L.) Merr.] growth stages can impact biological nitrogen fixation (BNF), increase yield and improve seed composition [protein, oil, and amino acid (AA) concentrations]. The objectives of this study were to evaluate the effect of different soybean inoculation strategies (seed coating and additional soil inoculation at V4 or R1) on: (i) seed yield, (ii) seed composition, and (iii) BNF traits [nodule number and relative abundance of ureides (RAU)]. Soybean field trials were conducted in 11 environments (four states of the US) to evaluate four treatments: (i) control without inoculation, (ii) seed inoculation, (iii) seed inoculation + soil inoculation at V4, and (iv) seed inoculation + soil inoculation at R1. Results demonstrated no effect of seed or additional soil inoculation at V4 or R1 on either soybean seed yield or composition. Also, inoculation strategies produced similar values to the non-inoculated control in terms of nodule number and RAU, a reflection of BNF. Therefore, we conclude that in soils with previous history of soybean and under non-severe stress conditions (e.g. high early-season temperature and/or saturated soils), there is no benefit to implementing additional inoculation on soybean yield and seed composition.

## Introduction

Soybean is a major globally produced oilseed crop, with the United States responsible for 35% of the 342 million Mg global production^1^. Soybean is one of the largest sources of vegetable oil and protein for humans and a high-quality animal feed^2^. Soybean production is forecasted to grow by 55%, in contrast with the 100–110% expected demand increase by 2050^3^. Therefore, increasing overall productivity via implementation of best management technologies is a research priority from a food security goal.

Seed composition refers to major constituents including protein, oil, carbohydrates, isoflavones, and mineral concentration that determine seed nutritional value^4^. Soybean meal has great economic importance in human foods and as a source of high-quality protein in animal diets^5^. Thus, an accurate description of soybean seed composition is a necessary requirement for the food and feed industry. Specifically, characterizing amino acids (AAs) composition is an important factor for formulating balanced diets, with this requirement varying between animal species and within categories of the same specie^6^. Therefore, AAs are classified as essential (EAA; AAs with carbon skeletons not synthesized by animals), including arginine, cysteine, histidine, isoleucine, leucine, lysine, methionine, phenylalanine, threonine, tryptophan, and valine; and non-essential AA (NEAA; AAs that can be synthesized by animals) including alanine, aspartic acid, glutamic acid, glycine, proline, serine, and tyrosine^5,7,8^. Moreover, quantifying the sulfur-containing AAs (cysteine and methionine) could be of particular interest for the food industry as soybean, as well as other legume species, is deficient in these AAs^9^. Variation in seed composition is affected by different physiological and biochemical mechanisms and may also be influenced by genotype (G), environment (E), management practices (MP), and their interactions (G × E × MP)^4,10^. Thus, understanding how these factors and their interactions impact seed composition is crucial for maintaining high yield and quality.

Soybean has a high nitrogen (N) requirement as it needs to uptake 80 kg N per Mg grain produced^11^. On average 50–60% and up to 90% of this N is provided through biological N fixation (BNF)^12,13^ by symbiotic soil bacteria, mainly *Bradyrhizobium japonicum*. Some studies indicated that soybean inoculation did not increase yield in sites where soybean has been previously grown^14–16^, due to the established symbiotic bacteria populations in the soil^14,17^. However, studies conducted in Brazil provide evidence of inoculation success in areas with high rhizobia population (10^3^–10^6^ cells g^−1^ of soil)^18–20^, opening a perspective for inoculation research in other geographic regions. A study conducted across 187 site-years in the US indicated that overall yield increase due to seed inoculation was 60 kg ha^−1^ (1.7%)^21^. Thus, these authors indicated that seed inoculation is a profitable practice, providing economic benefits.

Different inoculation strategies (here defined as the combination of timing and application form) could be used. Soybean inoculation is typically done by coating the seeds with bacteria cells before planting. However, a recent study conducted in Brazil indicated that additional inoculation to the soil at different soybean stages increased nodulation and, in some situations, seed yield^22^. Senescence of nodules occurs at around full pod formation (R4 stage) and, consequently, BNF also declines at this stage^23,24^, suggesting a shortage of N during key pod filling process. However, the formation of new nodules via additional inoculation could offset this shortcoming in N supply. Thus, we hypothesized that additional inoculation can improve soybean N nutrition promoting yield increase and improving seed protein composition (greater protein and AAs concentrations).

The aim of this study was to evaluate the effect of different soybean inoculation strategies (i.e. seed coating and additional soil inoculation at V4 or R1) on: (i) seed yield, (ii) seed composition (protein, oil, and AAs), and (iii) BNF traits (nodule number and relative abundance of ureides, RAU) as reflection on the BNF process.

## Results

### Weather conditions

**Figure 1:**
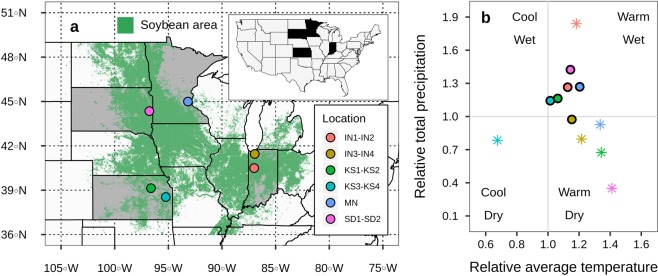
Map of the six sites (total of 11 soybean environments) (**a**) and weather characterization (**b**): temperature and precipitation relative to the average of the last 30 years (1987–2017) for each site and period (asterisks represent the first month after planting and circles represent the average of the entire crop growing season). Each location represented in the map refers to two trials planted in the same site, except for MN, Minnesota. IN = Indiana, KS = Kansas, and SD = South Dakota.

Across the 11 environments, the average seasonal temperature was ~10% higher than the 30 years normal (1987–2017) (Fig. 1b). Total precipitation during the growing season was similar to the 30 years normal for IN3, IN4, KS1, KS2, KS3, and KS4 sites, and roughly 30% greater than the 30 years normal for IN1, IN2, MN, SD1, and SD2 sites (Fig. 1b). For the weather conditions during the first 30 days after planting, when nodulation establishment occurs, temperature averaged ~25% higher than the 30 years normal at all the sites except at KS3 and KS4, where temperatures were ~30% below to the 30 years normal (Fig. 1b). Precipitation during the first 30 days of the cropping season was 80% greater than to the 30 years normal at IN1 and IN2, still without reflecting flooding problems, and 40% dryer than to the 30 years normal at KS1, KS2, SD1, and SD2 (Fig. 1b).

### Seed yield

**Figure 2:**
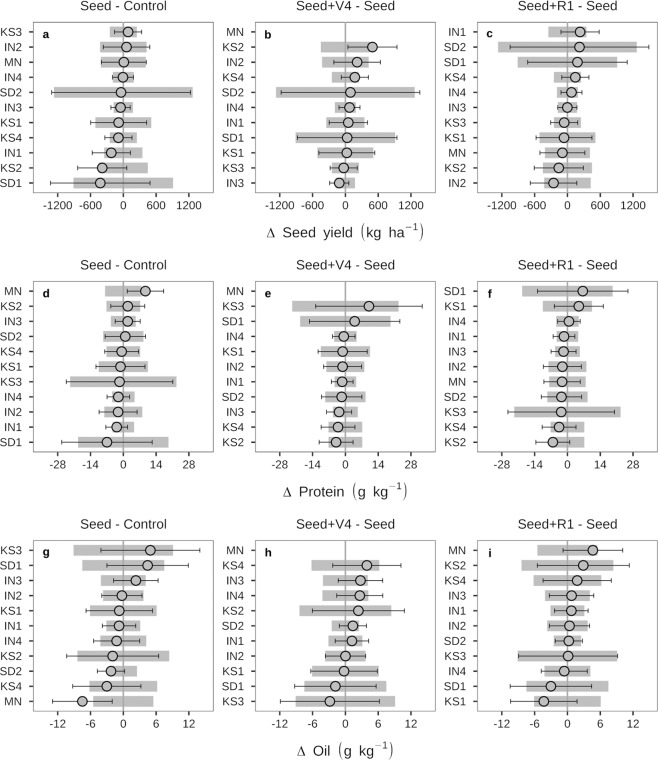
Difference between seed inoculation and non-inoculated control, and additional inoculation at fourth trifoliate (V4) and beginning of flowering (R1) for seed yield (**a**–**c**), protein (**d**–**f**), and oil concentration (**g**–**i**) in soybean seeds from 11 environments evaluating different soybean inoculation strategies. Error bars for each treatment difference and shaded bars indicate the 95% confidence interval for the baseline treatment in each of the evaluated sites. IN = Indiana, KS = Kansas, SD = South Dakota, and MN, Minnesota.

Average yield across all environments and treatments was 3437 kg ha^−1^, ranging from 1854 kg ha^−1^ at KS1 to 4387 kg ha^−1^ at IN2 (Supplementary Table S1). Seed inoculation did not increase seed yield in any environment (Fig. 2a). Likewise, additional soil inoculation at either V4 or R1 growth stages did not affect seed yield relative to the traditional inoculation to the seed, applied immediately prior to planting (Fig. 2b,c).

### Seed composition

Protein concentration was ~420 g kg^−1^ on average across all environments and ranged from 385 g kg^−1^ at IN4 to 451 g kg^−1^ at KS4 (Supplementary Table S1). Average oil concentration across all environments was ~210 g kg^−1^, ranging from 200 g kg^−1^ at KS1 to 231 g kg^−1^ at IN3 (Supplementary Table S1). Seed, as well as additional soil inoculation at V4 or R1, did not affect protein or oil concentration of soybean seeds (Fig. 2d–i). Inoculation strategies did not affect AAs concentration, except in nine out of the total of 198 AAs x environments combinations, when significant differences were lower than 3% between treatments, but without any clear trend of treatment effect across environments (Supplementary Table S2).

### Relationship between seed composition constituents

We generated three groups of AAs based on the slope of the relationship between relative AA and relative protein concentrations in the seed. Tryptophan was the only AA not related to protein concentration. For three AAs (arginine, glutamic acid, and cysteine) the slope was > 1, especially for cysteine (slope = 1.7); for two AAs (serine and threonine) the slope was equal to one; and for the remaining 12 AAs the slope was < 1, being the lowest value for isoleucine (slope = 0.58) (Supplementary Fig. S1).

**Figure 3:**
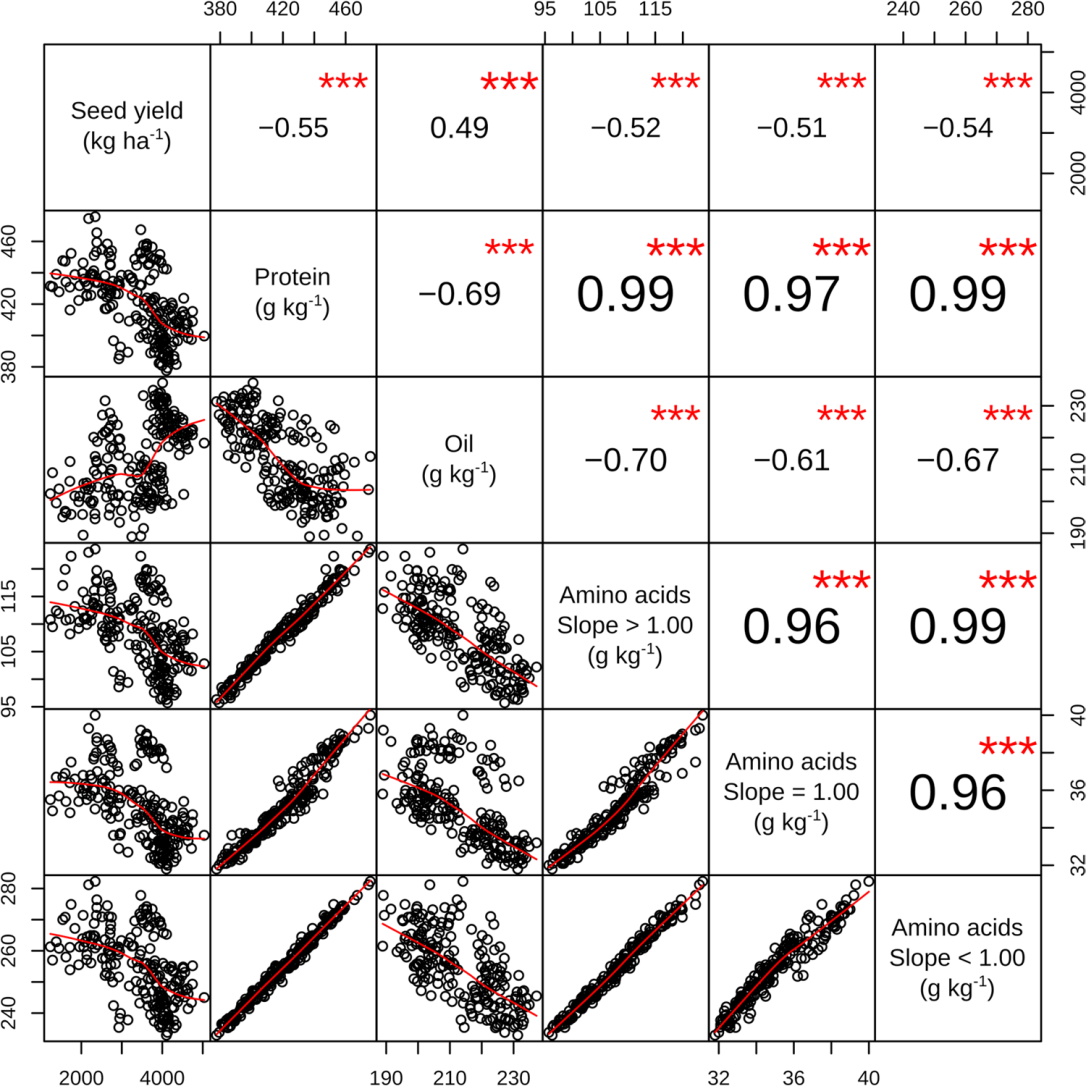
Correlation scatterplot of Pearson correlation between seed yield, protein, oil, and amino acids concentrations grouped based on their relationship with protein content [slopes greater (arginine, cysteine, and glutamic acid), equal (serine and threonine) or lower than one (alanine, aspartic acid, glycine, histidine, isoleucine, leucine, lysine, methionine, phenylalanine, proline, tryptophan, tyrosine, and valine); Supplementary Fig. S1] in 11 environments evaluating different soybean inoculation methods. The top half of the table shows the correlation coefficients and their significance (***indicates p < 0.001) and the bottom half shows the scatterplot with a red fitted line.

Pearson’s correlation between yield and seed composition constituents (protein, oil, and AAs groups) indicated that protein, as well as AAs groups, were negatively correlated with seed yield, while oil was positively correlated with seed yield (Fig. 3). Protein and AAs groups were positively and highly correlated with one another (r > 0.96), but both were negatively correlated with oil concentration (r = −0.69 and −0.61 to −0.70, respectively) (Fig. 3).

### BNF parameters

**Figure 4:**
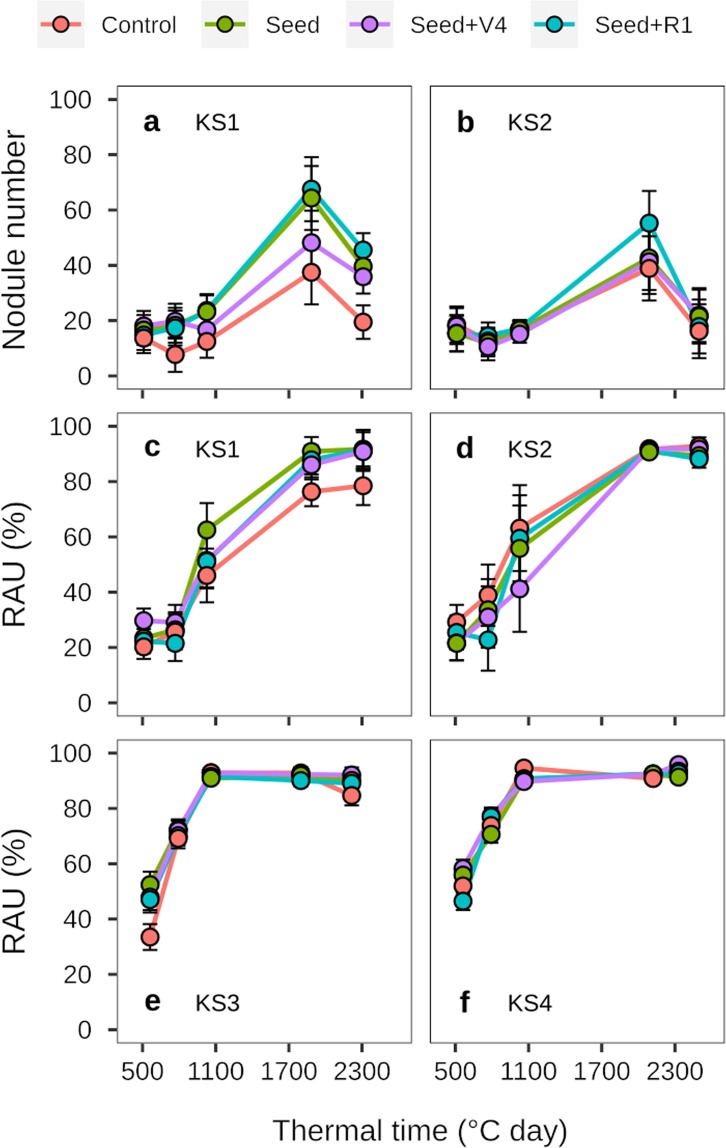
Nodule number per plant (**a**,**b**) and relative abundance of ureides (RAU) (**c**–**f**) at different soybean growth stages for the treatments: control (non-inoculated), seed inoculation at planting, and additional inoculations at V4 (fourth trifoliate) and R1 (beginning of flowering) in four environments (KS1, KS2, KS3, and KS4). Error bars indicate the standard deviation for each data point (mean). KS = Kansas.

For the environments in KS, nodule number per plant was not affected by inoculation treatment at any stage (Fig. 4a). Moreover, nodule number dynamics through the growing season was approximately constant from V4 to R2 (avg. 16 nodules per plant at 0–20 cm depth), peaked at R6 (avg. 50 nodules per plant) and deceased at R7 (avg. 27 nodules per plant) in both environments (KS1 and KS2; Fig. 4a,b).

None of the inoculation strategies affected BNF, assessed via determination of RAU, at any stage and at any of the sites (Fig. 4c–f; Table 1). For the sites in KS, RAU increased as the growing season progressed and reached a maximum (~91%) at R2 (KS3 and KS4) or R6 (KS1 and KS2). Across all the growing season and treatments, RAU averaged 57% for KS1 and KS2 and 79% for KS3 and KS4 (Fig. 4c–f). This difference between sites could be related to the tillage system, as conventional tillage was used at KS1 and KS2, promoting N mineralization, which could decrease BNF, while no-till was used at KS3 and KS4 (Table 2).

**Table 1:** Relative abundance of Ureide-N (RAU) at the R7 growth stage (mean ± SD; n = 3) for the treatments: control (non-inoculated), seed inoculation at planting, and additional inoculations at V4 (fourth trifoliate) and R1 (beginning of flowering) in seven environments (Env.) (KS1, KS2, KS3, KS4, MN, SD1, and SD2).

Env.	p-value	Treatment	RAU
			(%)
KS1	0.49	Control	78.5 ± 23.7
		Seed	91.7 ± 1.8
		Seed + V4	90.8 ± 3.4
		Seed + R1	91.5 ± 3.3
KS2	0.72	Control	92.8 ± 1.9
		Seed	89.4 ± 3.5
		Seed + V4	91.7 ± 2.5
		Seed + R1	88.2 ± 9.9
KS3	0.46	Control	84.5 ± 9.2
		Seed	90.1 ± 4.4
		Seed + V4	92.1 ± 3.0
		Seed + R1	89.1 ± 2.7
KS4	0.15	Control	92.8 ± 3.3
		Seed	91.3 ± 4.7
		Seed + V4	95.8 ± 1.3
		Seed + R1	92.7 ± 3.8
MN	0.55	Control	77.8 ± 4.9
		Seed	75.0 ± 13.4
		Seed + R1	81.8 ± 3.6
SD1	0.80	Control	73.1 ± 8.9
		Seed	77.0 ± 8.7
		Seed + V4	74.8 ± 6.9
		Seed + R1	71.4 ± 8.2
SD2	0.31	Control	64.7 ± 16.8
		Seed	73.5 ± 6.2
		Seed + V4	74.2 ± 1.8
		Seed + R1	79.6 ± 11.1

**Table 2:** Location (State, latitude (Lat.), and longitude (Long.)), soil characteristics (soil type, pH (1:2.5), available phosphorus (P), and soil organic matter (SOM) at 0–20 cm soil depth), and crop management (variety, maturity group, seeding rate, row spacing, previous crop, and tillage system) in 11 soybean environments (Env.).

Env.	Location			Soil characteristics				Crop management						
	State^§^	Lat.	Long.	Soil type	pH	P (mg kg^−1^)	SOM (g kg^−1^)	Variety	Maturity group	Planting date	Seeding rate (seeds ha^−1^)	Row spacing (m)	Previous crop	Tillage system
IN1	IN	40.4965	−86.9995	Typic Endoaquoll	6.4	34.6	34	AG24X7	2	24-May	345,800	0.38	corn	conventional
IN2	IN	40.4965	−86.9995	Typic Endoaquoll	6.4	34.6	34	AG34X6	3	24-May	345,800	0.38	corn	conventional
IN3	IN	41.4481	−86.9416	Typic Argiaquoll	6.2	31.8	37	AG24X7	2	25-May	345,800	0.38	corn	conventional
IN4	IN	41.4481	−86.9416	Typic Argiaquoll	6.2	31.8	37	AG34X6	3	25-May	345,800	0.38	corn	conventional
KS1	KS	39.1369	−96.6185	Fluventic Hapludoll	6.1	52.6	22	AG30X8	3	27-Apr	444,600	0.76	soybean	conventional
KS2	KS	39.1369	−96.6185	Fluventic Hapludoll	6.1	52.6	22	AG45X6	4	27-Apr	444,600	0.76	soybean	conventional
KS3	KS	38.538	−95.2401	Abruptic Argiaquoll	6.1	13.7	40	AG30X8	3	10-May	370,500	0.76	corn	no-till
KS4	KS	38.538	−95.2401	Abruptic Argiaquoll	6.1	13.7	40	AG45X6	4	10-May	370,500	0.76	corn	no-till
MN	MN	44.9942	−93.1731	Typic Hapludoll	6.2	16.4	36	AG14X7	1	11-May	346,000	0.76	corn	conventional
SD1	SD	44.3472	−96.7714	Aeric Calciaquoll	6.9	32.3	47	AG24X7	2	18-May	346,000	0.76	corn	conventional
SD2	SD	44.3472	−96.7714	Aeric Calciaquoll	6.9	32.3	47	AG11X8	1	18-May	346,000	0.76	corn	conventional

^§^IN, Indiana; KS, Kansas; MN, Minnesota; SD, South Dakota.

## Discussion

Overall, seed yields were in line with values previously reported in the evaluated regions^16,21,25^. The lowest yields (KS1 and KS2) were associated with conditions during the growing season warmer and drier than the 30 years normal (Fig. 1b)^26^. In addition, the utilization of conventional tillage on these environments negatively impacted the conservation of water in these soils^27,28^.

Classifying the AAs based on the magnitude of their changes with the variation in protein concentration provides practical and valuable information. Only two AAs (serine and threonine) varied in a similar proportion with protein, while most of the AAs increased their concentrations in lower proportion relative to increases in protein, except for arginine, cysteine, and glutamic acid (Fig. S1). The latter reflects the complexity on uniformly increasing AAs concentration in soybean seeds. In this line, Medic *et al*.^8^ and Pfarr *et al*.^29^ reported that most of the EAAs decreased as protein concentration increased, while glutamic acid and arginine increased in relative abundance. Our results showed that increases in protein produce a more than proportional increase in cysteine and close to a proportional increase in methionine, showing that increases in protein would partially overcome the low concentration of these deficient AAs.

It should be noted that in our study, variations in protein concentration were due to different conditions across environments (e.g. cultivar, management factors, and environmental characteristics), and not due to inoculation strategies. Thus, it is possible that other management practices (such as fertilization) or environmental factors, could differentially affect deposition of protein and AAs in soybean seeds. For example, Gayler and Sykes^30^ reported that S deficiency in soybean depressed cysteine and methionine concentration without affecting seed protein concentration. In addition, Pfarr *et al*.^29^ documented that different environmental stress conditions affected AAs balance; therefore, variations in AAs could be not directly predicted by changes in protein concentration. A limitation of this study regarding the classification of the relationship between AAs and protein is that it is only valid for grouping classes of AAs with the data set collected from this research project, and extending the application to other datasets would require independent validation.

Seed protein and oil concentration ranged within the values reported for modern soybean varieties^4,10,25,29^. Seed yield and oil concentration were negatively related to protein and AAs concentration (Fig. 3). Negative relationships between yield or oil and protein or AAs have been extensively reported^10,29,31,32^. This is because a low seed set (usually related to low yields) produces high N reserves per pod (generating high protein and AAs concentration) and the low C/N ratio in developing seeds produces a lower oil concentration, as increases in protein occur at the expense of oil and carbohydrates^29,32^.

Overall, seed inoculation and additional soil inoculation at V4 or R1 did not affect any of the parameters evaluated (seed yield, protein, oil, and AAs concentration in seeds), even when using two different commercially available inoculants (KS1, KS2, KS3, and KS4 vs. IN1, IN2, IN3, IN4, MN, SD1 and SD2). Our results related to seed yield are in agreement with the outcomes reported by Ham *et al*.^15^, Ellis *et al*.^14^, and de Bruin *et al*.^16^, but opposite (due to the lack of response on seed yield to inoculation) as those reported by Albareda *et al*.^17^ and Ruiz Diaz *et al*.^25^, and to those related to the evaluation of additional soil inoculation^22,33^.

No previous studies have assessed the effect of inoculation strategies on soybean seed composition. We hypothesized that this practice could increase soybean protein and AAs concentration in seeds. However, our data did not support our hypothesis. Some studies observed increases in seed protein concentration due to seed inoculation^17,34^, while others did not observe changes in protein concentration due to this practice^22,25^.

In the studies where inoculation increased protein concentration, increases in seed yield were also observed, indicating that N limited both yield and protein formation. However, no attempts were done previously to relate yield or protein response to inoculation with BNF. In this study, BNF was indirectly quantified via RAU method but presenting as a main limitation that this technique provides a single-point measurement of the N coming from BNF at the moment of sampling. Under the conditions evaluated in this study, none of the inoculation strategies affected nodule number or RAU, explaining the lack of yield and seed composition response to inoculation. This fact could be explained by many factors, among them:

I. Soil rhizobia population: Soils where studies responded to inoculation were devoid of soybean-nodulating rhizobia^17^ or did not have history of soybean production^25,34^. Therefore, lack of response can be partially connected to the evaluation at sites with previous history of soybean production that have a naturalized population of rhizobia in the soil^14,35^. Related to this, Hungria *et al*.^36^ reported that different inoculation strategies increased nodule number in soils where soybean was grown for the first time, but there was no effect of inoculation on nodulation in a soil with a *Bradyrhizobium* population already established. Moreover, Thies *et al*.^35^ indicated that the response to inoculation was inversely related to the soil rhizobia population.
II. Stress conditions: Studies conducted in Brazil reported a positive effect of additional inoculation on nodule number and seed yield, and these positive responses were associated with stressful growing conditions during the nodulation process, such as drought, heat, and soil acid pH^22,33^. However, even though low precipitation was observed at KS1, KS2, SD1, and SD2 during the period of nodule establishment, no responses to additional inoculation were observed in our study. On the other hand, soils of trials conducted in Brazil had a soil pH between 4.6 and 5.6, while our trials soil pH ranged from 6.1 to 6.9. Soil acidity reduces rhizobia survival in the soil, and also produces failure in nodulation, especially early in the infection process^37^. In this scenario, additional soil inoculation could have beneficial effects on soybean productivity.
III. Soil organic matter: Increases in N mineralization potential and consequently inorganic N availability are expected in soils with high soil organic matter content if the soil moisture is at optimum levels^38^. Therefore, a lower contribution of BNF on soybean N nutrition may be expected in soils with high organic matter^39^. According to this, Thies *et al*.^35^ indicated that N mineralization potential of the soil was a relevant variable to consider when predicting yield response to inoculation of legumes. Likewise, de Bruin *et al*.^16^ and Leggett *et al*.^21^ indicated low probability of yield response to inoculation in soils with high organic matter content. For this reason, it is likely that in our trials, soil organic matter (from 22 to 47 g kg^−1^; Table 2) was high enough to supply N to soybean through mineralization, decreasing the dependency on BNF. In contrast, the soil organic matter concentration at the responsive sites in Brazil was between 5 and 18 g kg^−1^ ^22,33^, which would limit the amount of N available for mineralization and increase the dependency on BNF. Additionally, excessive precipitation at the Brazilian sites would increase the likelihood of nitrate leaching, further reducing soil N availability.
IV. Yield environment: It was reported that N limitation in soybean could be especially prevalent in high yielding environments (>4500 kg ha^−1^)^11,12,40^. Greater plant N requirements lead to an increase in the need for N, and consequently greater responses to inoculation^21^. Under this situation, additional soil inoculation at V4 or R1 could help satisfy N requirements. However, maximum seed yield in our study was ~4400 kg ha^−1^ (IN2, Table S1).

The lack of response in seed yield and seed composition to inoculation strategies could be explained by the quantity and type of rhizobia bacteria population in the evaluated sites^35^. However, due to the lack of information, we are not able to confirm this hypothesis, even though inoculated soybean crops were part of the crop rotation in the evaluated sites, which provides evidence of rhizobia presence in the soils. Therefore, future studies should consider the abundance and speciation determination of rhizobia bacteria in the soil. Moreover, strain identification would also be valuable, as there are differences in BNF efficiency among strains^41,42^. In addition, information on rhizobia bacteria survivability after soil inoculation at V4 and R1 would be valuable to help in explaining the lack of response to additional soil inoculation.

As previously discussed, N limitations were reported in high yielding environments^11,12,40^. Therefore, it may be possible to detect differences across inoculation strategies in high-yielding environments (>4500 kg ha^−1^), which should be tested in future studies.

Lastly, stresses such as soil salinity, extreme temperatures, and moisture (flooding or drought) can influence rhizobia survival, nodulation, and negatively impact the BNF process^37,43,44^. For example, the nodule initiation process is very sensitive to salt or osmotic stress, with extreme temperatures affecting root-hair infection and nodule structure^37^. Neither extreme dryness, flooding, nor heat or other extreme stress conditions were reported early-season in our sites. Nonetheless, we acknowledge that potential early-season stress conditions can have a large impact on the nodulation process. Therefore, impact of stress on late-season inoculation should be further investigated.

## Conclusions

Soybean seed yield and composition (protein, oil, and AAs concentration) were not affected by any of the inoculation strategies. This was explained by the lack of response to inoculation on nodule number and RAU. It is likely that the lack of severe stress conditions during the nodulation process (early during the crop establishment) and soils with previous soybean in the rotation or viable rhizobia populations create conditions in which seed or additional in-season soil inoculations, at V4 or R1 growth and development stages, did not produce any benefit on seed yield and composition.

## Methods

### Sites, treatments and experimental design

Soybean field trials were located in six sites where two varieties were tested in five of them generating 11 different environments. Trials were conducted during 2018 growing season in different regions of the US (Indiana, IN1-IN4; Kansas, KS1-KS4; Minnesota, MN; and South Dakota, SD1-SD2) (Table 2; Fig. 1a). The sites were located within the top 10 US states on soybean production and covered a wide range of soil and climatic conditions. In each environment, a randomized completed block design with four to six replicates (plot size 3 × 8 m) was used to evaluate four treatments: (i) control without inoculation, (ii) seed inoculation, (iii) seed inoculation + soil inoculation at V4 (fourth trifoliate^45^;), and iv) seed inoculation + soil inoculation at R1 (beginning of flowering). All the treatments were evaluated at all sites, except treatment iii at the MN site, where soil inoculation at V4 was not possible to be done due to operational problems. *Bradyrhizobium japonicum* inoculant was used for all the inoculation strategies. For seed coating, a dose of 2–2.8 ml kg seed^−1^ applied to the seed before planting, following the product´s label [VAULT HP plus Integral (BASF Chemical Corporation, New Jersey, USA) at KS1, KS2, KS3, and KS4; and America´s Best Inoculant (Advanced Biological Marketing, Ohio, USA) at IN1, IN2, IN3, IN4, MN, SD1 and SD2]. For soil inoculation, the same products as for seed coating were used with a rate of 0.25–1.25 L ha^−1^, based on the recommendation from the manufacturer, diluted in water at a final volume of 200–280 L ha^−1^, and applied surface banded to the soil.

All the environments were located in an area where inoculated soybean was grown in the previous 10 years, except at KS3 and KS4. Those two environments were conducted in soil with a continuous corn (*Zea mays* L.) monoculture over the last 14 years, although soybean was part of the crop rotation earlier. All the environments included seed treated with fungicide, except at MN. Weeds were controlled by the herbicide glyphosate [N-(phosphonomethyl)glycine] applied three weeks after planting at a rate of 1.5 kg a.i. ha^−1^. Crop management followed the best recommended practices for each environment and are described in Table 2.

### Measurements and sampling

After physiological maturity (R8^45^,) plots were harvested (11.6 m^2^), and yield was adjusted to 130 g kg^−1^ moisture content. Seed samples were collected from each plot to analyze for seed composition. Protein, oil, and AAs concentration were determined via near-infrared spectroscopy using a Perten DA7250 diode array instrument (Perten Instruments, Springfield, IL) equipped with calibration equations developed by the University of Minnesota in cooperation with Perten Instruments. This method provides concentration of 18 AAs, but does not differentiate between glutamine or glutamate and aspartate or asparagine. Thus, glutamic acid estimates the sum of glutamine and glutamate and aspartic acid estimates the sum of aspartate and asparagine^29^. Seed composition traits were also adjusted to 130 g kg^−1^ moisture content.

For characterizing growing conditions in each site, weather data (precipitation and daily mean temperature) were obtained from Climate Engine^46^ (Fig. 1a,b). Relative total precipitation and relative average temperature were calculated as the ratio between the 2018 growing season conditions and the last 30 years average (1987–2017) of each site.

### Nodulation and BNF traits

In four out of the 11 environments (KS1, KS2, KS3, and KS4) detailed measurements were collected to evaluate nodulation and BNF. At KS1 and KS2, the total number of nodules (viable and not) per plant was quantified at different growth stages [V4; V7 (seventh trifoliate); R2 (full flowering), R6 (full seed), and R7 (beginning maturity)]. At each of these growth stages five consecutive plants per plot were carefully removed from the soil (0–20 cm soil depth) and washed with water to separate the soil from the roots and nodules.

Main stem samples of ten (V4 and V7) or five (R2, R6, and R7) plants per plot were collected at the same stages as for nodule counts at four sites (KS1, KS2, KS3, and KS4) for the relative abundance of ureides-N (RAU) determination. For the same purpose, main stem samples of five plants per plot were collected in sites MN, SD1, and SD2 at R7 growth stage. Stems were dried and ground (1-mm mesh). The RAU determination in the main stems followed the procedure of Hungria and Araujo^47^. The RAU was calculated as a function of ureide-N and nitrate-N molar concentration^48^:

$${\rm{RAU}}( \% )=\frac{4\ast {\rm{ureide}}\,{\rm{conc}}.}{[(4\ast {\rm{ureide}}\,{\rm{conc}}.)+{\rm{nitrate}}-{\rm{N}}\,{\rm{conc}}.]}\ast 100$$

### Statistical analysis

Mixed models were fitted for each variable (seed yield, protein, oil, and AA concentration, nodule number, and RAU) in each location using the “lme4” package^49^ within the R software^50^. The inoculation strategy was considered as a fixed effect, while the block was a random effect. For the Kansas locations (KS1, KS2, KS3, and KS4), with in-season measurements to characterize BNF, an analysis of variance (ANOVA) was implemented at each growth stage, also considering the block as a random component in the model. Before running the ANOVA, normality of the residuals and homogeneity of the variances were tested using Shapiro-Wilk and Levene’s test, respectively. Tukey test (5% significance) was performed for means comparison using the “multcomp” package^51^ within the R software.

Amino acids were grouped in order to simplify data visualization and discussion. The criteria were based on the relationship between each AA and protein. At first, all the AAs and protein were converted to a relative basis (relative to the minimum value over all the environments and treatments for each AA and protein concentration, respectively), and the slopes were compared to 1 (having relative protein on the *x* axis and each relative AA on the *y* axis). Amino acids were grouped into four classes: A) slope greater than 1, AAs that increase at a higher rate than protein; B) slope equal to 1, AAs following the same trend as protein; C) slope lower than 1, AAs increasing at lower rates than protein; and D) AAs without significant relationship with protein (non-significant slope). Soybean yield, protein, oil and the AAs concentrations from each class were evaluated in a correlation matrix (Pearson correlation), using the “PerformanceAnalytics” package^52^.

## Supplementary information

Supplementary information
