# Supplementary material for: Soybean yield, biological N2 fixation and seed composition responses to additional inoculation in the United States
Source: Sci Rep. 2019 Dec 27;9:19908. doi: 10.1038/s41598-019-56465-0 (PMC6934618; doi:10.1038/s41598-019-56465-0)
Supplement: Supplementary file 1 — Supplementary information [file 41598_2019_56465_MOESM1_ESM.docx]

**Soybean yield, biological N_2_ fixation and seed composition responses to additional inoculation in the United States**

Walter D. Carciochi, Luiz H. Moro Rosso, Mario A. Secchi, Adalgisa R. Torres, Seth Naeve, Shaun N. Casteel, Péter Kovács, Dan Davidson, Larry C. Purcell, Sotirios Archontoulis, and Ignacio A. Ciampitti.

**Supplementary Table 1:** Seed yield and protein and oil concentrations in soybean seeds averaged across inoculation strategies (control without inoculation, seed inoculation, seed inoculation + soil inoculation at V4, and seed inoculation + soil inoculation at R1) and environment (Env.).

| Env. | **Treatment** | **Seed yield**  **(kg ha^-1^)** | **Protein**  **(g kg^-1^)** | **Oil**  **(g kg^-1^)** |
| --- | --- | --- | --- | --- |
| IN1 | Control | 4339 | 414.8 | 223.8 |
|  | Seed | 4328 | 412.0 | 223.4 |
|  | Seed + V4 | 4162 | 410.7 | 223.3 |
|  | Seed + R1 | 4108 | 410.6 | 222.6 |
| IN2 | Control | 4627 | 403.5 | 220.0 |
|  | Seed | 4411 | 401.3 | 219.8 |
|  | Seed + V4 | 4353 | 400.1 | 219.6 |
|  | Seed + R1 | 4157 | 399.1 | 219.6 |
| IN3 | Control | 3976 | 398.7 | 233.6 |
|  | Seed | 3925 | 397.1 | 231.6 |
|  | Seed + V4 | 3924 | 396.9 | 230.8 |
|  | Seed + R1 | 3813 | 396.1 | 228.5 |
| IN4 | Control | 4118 | 386.5 | 229.4 |
|  | Seed | 4117 | 385.0 | 228.0 |
|  | Seed + V4 | 4049 | 384.4 | 226.8 |
|  | Seed + R1 | 4042 | 383.7 | 226.2 |
| KS1 | Control | 1927 | 441.2 | 202.0 |
|  | Seed | 1868 | 437.5 | 201.3 |
|  | Seed + V4 | 1841 | 436.3 | 201.1 |
|  | Seed + R1 | 1779 | 435.2 | 197.0 |
| KS2 | Control | 3017 | 434.1 | 203.1 |
|  | Seed | 2907 | 432.2 | 202.6 |
|  | Seed + V4 | 2522 | 430.2 | 202.1 |
|  | Seed + R1 | 2363 | 427.9 | 200.2 |
| KS3 | Control | 2608 | 451.3 | 220.3 |
|  | Seed | 2577 | 442.9 | 220.1 |
|  | Seed + V4 | 2549 | 441.3 | 217.3 |
|  | Seed + R1 | 2520 | 438.6 | 215.2 |
| KS4 | Control | 3750 | 452.7 | 203.8 |
|  | Seed | 3722 | 452.0 | 202.8 |
|  | Seed + V4 | 3666 | 448.9 | 201.6 |
|  | Seed + R1 | 3577 | 448.5 | 199.8 |
| MN | Control | 3582 | 431.8 | 210.1 |
|  | Seed | 3572 | 429.6 | 207.2 |
|  | Seed + V4 | nd ^φ^ | nd | nd |
|  | Seed + R1 | 3490 | 422.4 | 202.6 |
| SD1 | Control | 3765 | 406.7 | 213.5 |
|  | Seed | 3527 | 406.3 | 211.7 |
|  | Seed + V4 | 3377 | 403.7 | 210.5 |
|  | Seed + R1 | 3344 | 399.7 | 209.1 |
| SD2 | Control | 3493 | 419.0 | 202.5 |
|  | Seed | 3365 | 418.2 | 201.6 |
|  | Seed + V4 | 3314 | 417.5 | 200.5 |
|  | Seed + R1 | 3272 | 416.5 | 200.2 |

^φ^ nd, not determined.

**Supplementary Table 2:** Concentration of 18 amino acids in soybean seed in 11 environments (Env.) evaluating different soybean inoculation strategies (control without inoculation, seed inoculation, seed inoculation + soil inoculation at V4, and seed inoculation + soil inoculation at R1).

| **Env.** | **Treatment** | **Ala^§^** | **Arg** | **Asp** | **Cys** | **Glu** | **Gly** | **His** | **Ile** | **Leu** | **Lys** | **Met** | **Phe** | **Pro** | **Ser** | **Thr** | **Trp** | **Tyr** | **Val** |
| --- | --- | --- | --- | --- | --- | --- | --- | --- | --- | --- | --- | --- | --- | --- | --- | --- | --- | --- | --- |
|  |  | **- - - - - - - - - - - - - - - - - - - - (g kg^-1^)- - - - - - - - - - - - - - - - - - - -** | | | | | | | | | | | | | | | | | |
| IN1 | Control | 17.4 | 29.3 | 46.9 | 6.60 | 71.8 | 17.9 | 10.5 | **20.0 a^¥^** | 31.5 | 27.2 | 5.70 | **21.7 a** | 19.8 | 18.4 | 16.0 | 4.80 | 15.5 | 19.8 |
|  | Seed | 17.3 | 29.0 | 46.2 | 6.60 | 70.6 | 17.7 | 10.3 | **19.8 ab** | 31.1 | 27.0 | 5.60 | **21.5 ab** | 19.7 | 18.2 | 15.8 | 4.80 | 15.3 | 19.5 |
|  | Seed + V4 | 17.3 | 28.8 | 46.0 | 6.60 | 70.5 | 17.7 | 10.3 | **19.7 b** | 30.9 | 27.0 | 5.70 | **21.3 b** | 19.6 | 18.2 | 15.8 | 4.80 | 15.3 | 19.4 |
|  | Seed + R1 | 17.3 | 29.1 | 46.4 | 6.50 | 70.9 | 17.8 | 10.4 | **19.9 ab** | 31.1 | 26.9 | 5.60 | **21.6 ab** | 19.6 | 18.3 | 15.8 | 4.90 | 15.4 | 19.6 |
| IN2 | Control | 17.0 | 28.4 | 45.1 | 6.50 | 68.4 | 17.4 | 10.2 | 19.4 | 30.5 | 26.5 | 5.50 | 21.1 | **19.4 a** | 18.0 | 15.6 | **4.90 a** | 15.1 | 19.2 |
|  | Seed | 16.9 | 28.3 | 44.8 | 6.40 | 68.4 | 17.3 | 10.1 | 19.3 | 30.2 | 26.3 | 5.40 | 20.9 | **19.1 b** | 17.9 | 15.5 | **4.80 ab** | 15.0 | 19.0 |
|  | Seed + V4 | 16.9 | 28.2 | 44.7 | 6.40 | 68.1 | 17.2 | 10.1 | 19.2 | 30.3 | 26.4 | 5.50 | 20.9 | **19.2 ab** | 17.9 | 15.5 | **4.80 b** | 14.9 | 19.0 |
|  | Seed + R1 | 16.9 | 28.3 | 44.8 | 6.40 | 68.1 | 17.2 | 10.0 | 19.3 | 30.3 | 26.4 | 5.40 | 20.9 | **19.1 ab** | 17.9 | 15.5 | **4.80 ab** | 15.0 | 19.0 |
| IN3 | Control | 16.8 | 27.6 | 44.8 | 6.40 | 67.6 | 17.1 | 10.0 | 19.2 | 30.2 | 26.2 | 5.40 | 20.9 | 19.0 | 17.8 | 15.4 | 4.70 | 15.0 | 18.9 |
|  | Seed | 16.8 | 27.7 | 44.7 | 6.30 | 67.5 | 17.1 | 10.0 | 19.3 | 30.2 | 26.2 | 5.40 | 20.8 | 19.0 | 17.7 | 15.3 | 4.60 | 14.9 | 19.0 |
|  | Seed + V4 | 16.8 | 27.5 | 44.6 | 6.30 | 67.0 | 17.1 | 10.0 | 19.2 | 30.1 | 26.2 | 5.40 | 20.8 | 19.0 | 17.7 | 15.2 | 4.70 | 14.9 | 18.9 |
|  | Seed + R1 | 16.8 | 27.5 | 44.7 | 6.30 | 67.1 | 17.2 | 10.0 | 19.2 | 30.1 | 26.2 | 5.40 | 20.9 | 19.1 | 17.7 | 15.3 | 4.70 | 15.0 | 18.9 |
| IN4 | Control | 16.4 | 26.7 | 43.3 | 6.20 | 65.0 | 16.6 | 9.70 | 18.7 | 29.5 | 25.8 | 5.30 | 20.2 | 18.4 | 17.4 | 15.1 | 4.60 | 14.6 | 18.5 |
|  | Seed | 16.4 | 26.8 | 43.3 | 6.20 | 65.1 | 16.6 | 9.70 | 18.7 | 29.5 | 25.8 | 5.30 | 20.2 | 18.6 | 17.3 | 15.0 | 4.60 | 14.6 | 18.5 |
|  | Seed + V4 | 16.4 | 26.7 | 43.2 | 6.20 | 64.7 | 16.6 | 9.70 | 18.6 | 29.3 | 25.7 | 5.30 | 20.3 | 18.5 | 17.3 | 15.0 | 4.70 | 14.5 | 18.3 |
|  | Seed + R1 | 16.4 | 26.8 | 43.4 | 6.10 | 65.3 | 16.6 | 9.70 | 18.7 | 29.4 | 25.8 | 5.30 | 20.3 | 18.4 | 17.3 | 15.0 | 4.60 | 14.6 | 18.5 |
| KS1 | Control | 18.4 | 30.6 | 48.7 | 7.30 | 74.0 | 18.9 | 10.9 | 20.2 | 32.6 | 27.8 | 5.80 | 22.4 | 21.0 | 19.5 | 16.9 | 4.50 | 16.1 | 20.6 |
|  | Seed | 18.3 | 31.0 | 48.6 | 7.40 | 74.1 | 18.9 | 11.0 | 20.4 | 32.5 | 27.7 | 5.80 | 22.3 | 20.8 | 19.2 | 16.7 | 4.60 | 16.0 | 20.9 |
|  | Seed + V4 | 18.4 | 31.1 | 49.0 | 7.40 | 75.2 | 19.0 | 11.0 | 20.4 | 32.6 | 27.8 | 5.90 | 22.3 | 20.7 | 19.4 | 16.9 | 4.40 | 16.1 | 20.9 |
|  | Seed + R1 | 18.5 | 31.5 | 49.5 | 7.40 | 75.7 | 19.1 | 11.1 | 20.4 | 32.7 | 27.8 | 5.80 | 22.4 | 20.9 | 19.4 | 16.9 | 4.40 | 16.1 | 21.0 |
| KS2 | Control | 18.1 | 30.7 | 48.4 | 7.20 | 73.3 | 18.5 | 10.9 | 20.3 | 32.3 | 28.0 | 5.90 | 22.1 | 20.5 | 19.1 | 16.5 | 4.60 | 16.0 | 20.5 |
|  | Seed | 18.0 | 31.0 | 48.5 | 7.20 | 74.0 | 18.5 | 10.9 | 20.4 | 32.2 | 27.9 | 5.80 | 22.2 | 20.4 | 19.0 | 16.5 | 4.70 | 15.9 | 20.5 |
|  | Seed + V4 | 17.9 | 30.4 | 47.9 | 7.10 | 72.9 | 18.4 | 10.7 | 20.3 | 32.0 | 27.8 | 5.80 | 22.0 | 20.4 | 19.0 | 16.4 | 4.60 | 15.8 | 20.3 |
|  | Seed + R1 | 17.9 | 30.5 | 47.6 | 7.10 | 72.8 | 18.3 | 10.8 | 20.1 | 31.9 | 27.6 | 5.80 | 21.8 | 20.4 | 18.9 | 16.4 | 4.90 | 15.7 | 20.2 |
| KS3 | Control | 18.5 | 30.2 | 49.5 | 8.00 | 75.9 | 19.1 | **11.0 ab** | 20.2 | 33.0 | 28.2 | 6.20 | 22.2 | 20.7 | 20.1 | 17.4 | 4.80 | **16.7 ab** | 20.6 |
|  | Seed | 18.4 | 30.1 | 49.4 | 7.90 | 75.6 | 19.0 | **10.9 ab** | 20.2 | 32.9 | 28.2 | 6.10 | 22.1 | 20.6 | 20.3 | 17.4 | 4.80 | **16.7 ab** | 20.4 |
|  | Seed + V4 | 18.7 | 30.9 | 50.4 | 8.10 | 77.6 | 19.4 | **11.2 a** | 20.5 | 33.6 | 28.6 | 6.20 | 22.5 | 20.9 | 20.5 | 17.7 | 4.90 | **17.0 a** | 20.9 |
|  | Seed + R1 | 18.3 | 29.7 | 48.9 | 7.90 | 74.7 | 18.8 | **10.8 b** | 19.9 | 32.7 | 28.1 | 6.10 | 21.9 | 20.4 | 20.1 | 17.3 | 4.80 | **16.6 b** | 20.3 |
| KS4 | Control | 18.7 | 32.0 | 50.7 | 7.80 | 78.2 | 19.3 | 11.4 | 20.6 | 33.8 | 28.9 | 6.20 | 22.6 | 21.2 | 20.7 | 17.7 | 4.80 | 16.9 | 20.9 |
|  | Seed | 18.7 | 31.8 | 50.6 | 8.00 | 77.7 | 19.2 | 11.4 | 20.5 | 33.5 | 28.9 | 6.30 | 22.6 | 21.2 | 20.6 | 17.7 | 4.80 | 16.8 | 21.0 |
|  | Seed + V4 | 18.6 | 31.6 | 50.4 | 8.00 | 77.7 | 19.1 | 11.4 | 20.4 | 33.6 | 28.9 | 6.20 | 22.5 | 21.1 | 20.6 | 17.7 | 4.90 | 16.8 | 20.9 |
|  | Seed + R1 | 18.6 | 31.4 | 50.1 | 7.90 | 76.8 | 19.1 | 11.3 | 20.5 | 33.4 | 28.8 | 6.20 | 22.5 | 21.0 | 20.4 | 17.5 | 4.70 | 16.8 | 20.9 |
| MN | Control | 17.7 | 29.7 | 47.0 | 7.10 | 71.6 | 18.1 | 10.6 | 19.9 | 31.5 | 27.4 | **5.90 b** | 21.4 | 20.4 | 18.8 | **16.3 b** | 4.60 | **15.4 b** | 20.0 |
|  | Seed | 17.9 | 30.5 | 47.9 | 7.20 | 73.3 | 18.4 | 10.7 | 20.1 | 32.1 | 27.9 | **6.00 ab** | 21.7 | 20.7 | 19.0 | **16.6 a** | 4.50 | **15.6 a** | 20.4 |
|  | Seed + V4 | nd^φ^ | nd | nd | nd | nd | nd | nd | nd | nd | nd | nd | nd | nd | nd | nd | nd | nd | nd |
|  | Seed + R1 | 17.9 | 30.3 | 47.8 | 7.20 | 73.2 | 18.4 | 10.7 | 19.9 | 32.0 | 27.7 | **6.10 a** | 21.6 | 20.7 | 19.1 | **16.6 a** | 4.60 | **15.7 a** | 20.2 |
| SD1 | Control | 17.0 | 28.7 | 45.8 | 6.80 | 69.9 | 17.5 | 10.3 | 19.2 | 30.5 | 26.6 | 5.70 | 20.9 | 19.3 | 18.3 | 15.8 | 4.70 | 15.1 | 19.2 |
|  | Seed | 16.9 | 27.8 | 45.1 | 6.80 | 68.3 | 17.4 | 10.0 | 18.9 | 30.0 | 26.4 | 5.70 | 20.5 | 18.8 | 18.1 | 15.8 | 4.70 | 14.9 | 18.8 |
|  | Seed + V4 | 17.0 | 28.2 | 45.5 | 6.80 | 69.4 | 17.5 | 10.3 | 19.2 | 30.5 | 26.5 | 5.70 | 20.7 | 19.3 | 18.2 | 15.8 | 4.80 | 15.1 | 19.2 |
|  | Seed + R1 | 17.1 | 28.4 | 45.6 | 6.80 | 69.5 | 17.5 | 10.2 | 19.1 | 30.6 | 26.7 | 5.80 | 20.7 | 19.2 | 18.3 | 16.0 | 4.80 | 15.1 | 19.3 |
| SD2 | Control | 17.4 | 29.8 | 46.7 | 6.80 | 71.4 | 17.8 | 10.6 | 19.5 | 31.2 | 27.1 | 5.70 | 21.2 | 19.8 | 18.7 | 16.2 | 4.90 | 15.4 | 19.5 |
|  | Seed | 17.5 | 30.0 | 46.9 | 6.80 | 71.8 | 17.9 | 10.6 | 19.7 | 31.5 | 27.3 | 5.80 | 21.5 | 19.8 | 18.7 | 16.3 | 4.90 | 15.5 | 19.9 |
|  | Seed + V4 | 17.4 | 30.0 | 46.6 | 6.70 | 71.4 | 17.8 | 10.5 | 19.6 | 31.2 | 27.1 | 5.70 | 21.3 | 19.8 | 18.7 | 16.2 | 4.80 | 15.3 | 19.8 |
|  | Seed + R1 | 17.4 | 30.1 | 46.7 | 6.80 | 71.6 | 17.9 | 10.6 | 19.7 | 31.3 | 27.1 | 5.70 | 21.5 | 20.1 | 18.7 | 16.1 | 4.90 | 15.4 | 19.8 |

**^§^** Ala, alanine; Arg, arginine; Asp, aspartic acid; Cys, cysteine; Glu, glutamic acid; Gly, glycine; His, histidine; Ile, isoleucine; Leu, leucine; Lys, lysine; Met, methionine; Phe, phenylalanine; Pro, proline; Ser, serine; Thr, threonine; Trp, tryptophan; Tyr, tyrosine; Val, valine.

**^¥^** Different letters in the same amino acid and environment indicate differences between inoculation treatments at P < 0.05 using Tukey test.

^φ^ nd, not determined


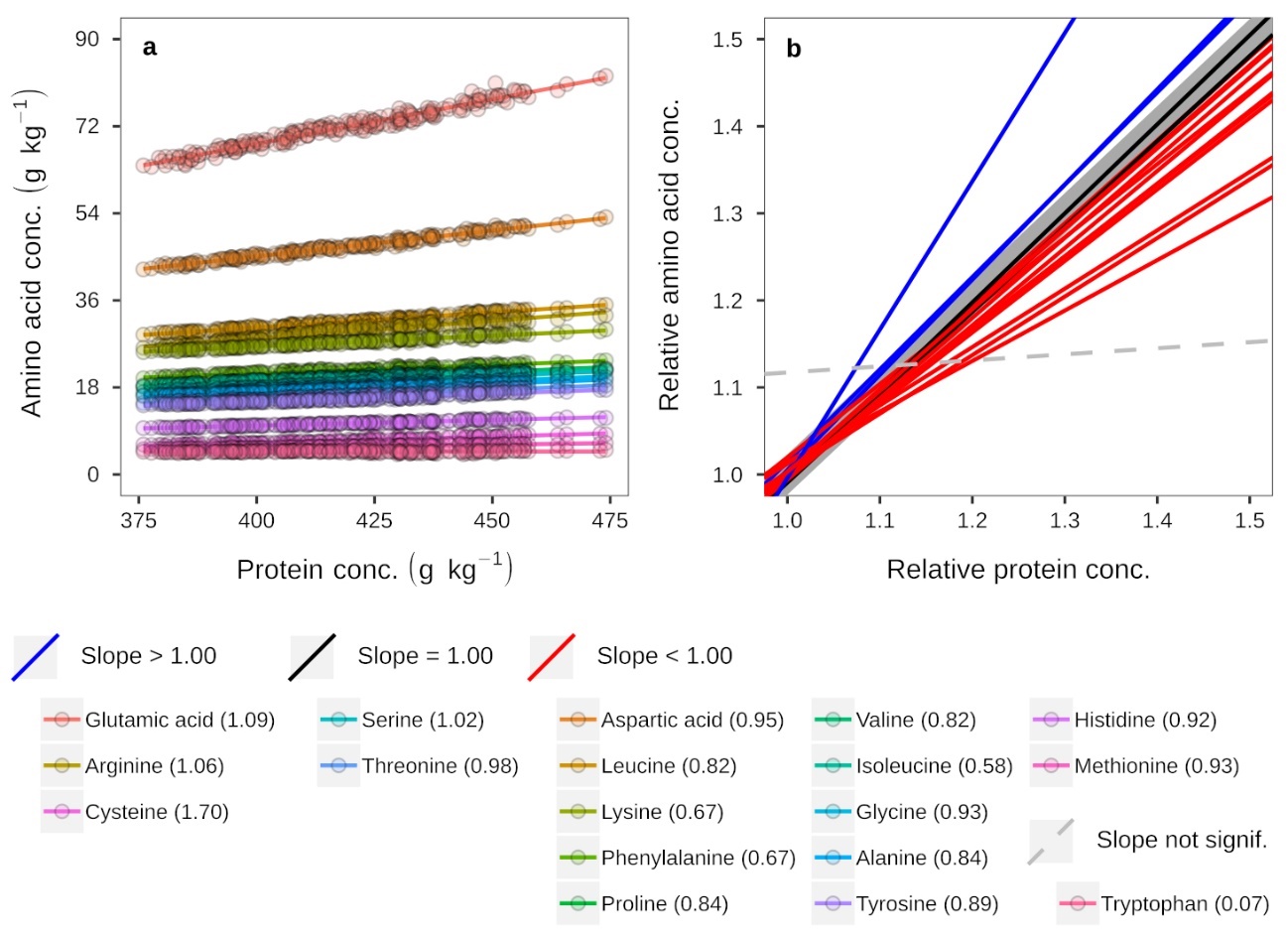


**Supplementary Figure 1.** Relationship between amino acids and protein concentrations (a), and relative amino acids and relative protein concentrations (b) in 11 environments evaluating different soybean inoculation strategies. Values between brackets indicate the slope of the relationship showed in panel B.
